# A Giant Duodenal Leiomyoma Showing Increased Uptake on 18F-Fluorodeoxyglucose Positron Emission Tomography

**DOI:** 10.1155/2018/7827163

**Published:** 2018-09-09

**Authors:** Keisuke Nonoyama, Hidehiko Kitagami, Akira Yasuda, Shiro Fujihata, Minoru Yamamoto, Yasunobu Shimizu, Moritsugu Tanaka

**Affiliations:** Department of Gastroenterological Surgery, Kariya Toyota General Hospital, Kariya, Japan

## Abstract

**Background:**

Although 18F-fluorodeoxyglucose positron emission tomography (FDG-PET/CT) is now widely used in their differential diagnosis, it is sometimes difficult to distinguish between benign and malignant diseases.

**Case Presentation:**

A 44-year-old woman was found to have abnormalities on health screening. Magnetic resonance imaging for detailed examination showed an intra-abdominal tumor measuring 12 cm in the major axis near the cranial end of the uterus. Upper gastrointestinal tract endoscopy showed a tumor with an ulcer in the third part of the duodenum, involving half the circumference. Heterogeneous uptake was observed within the tumor on FDG-PET/CT. Based on these findings, the patient underwent surgery for suspected primary malignant lymphoma of the duodenum or gastrointestinal stromal tumor. Laparotomy revealed a 12 cm tumor in the third part of the duodenum. Partial duodenectomy and end-to-end duodenojejunostomy were performed. Pathological findings showed a solid tumor growing from the muscle layer of the duodenum to outside the serous membrane; based on immunostaining, it was diagnosed as a leiomyoma.

**Conclusions:**

Duodenal leiomyomas are originally benign; to date, there have been no reports of uptake in duodenal leiomyomas on FDG-PET/CT; therefore, our case is rare. Leiomyomas should be considered in the differential diagnosis of duodenal neoplastic diseases.

## 1. Background

18F-fluorodeoxyglucose positron emission tomography (FDG-PET/CT) is now widely used in the differential diagnosis of benign and malignant diseases [1]. In general, no increased uptake is observed in duodenal leiomyomas, which are benign tumors. We herein report our experience with a case of a giant duodenal leiomyoma showing increased uptake on FDG-PET/CT.

## 2. Case Presentation

A 44-year-old woman was diagnosed with iron deficiency anemia but showed no abnormalities on gastrointestinal tract endoscopy 5 years prior to the current presentation. A blood test for health screening showed anemia with hemoglobin 7.6 g/dL, and uterine fibroids were suspected on abdominal ultrasonography. She was diagnosed as having an intra-abdominal tumor on magnetic resonance imaging (MRI) for detailed examination and was referred to our hospital.

**Figure 1:**
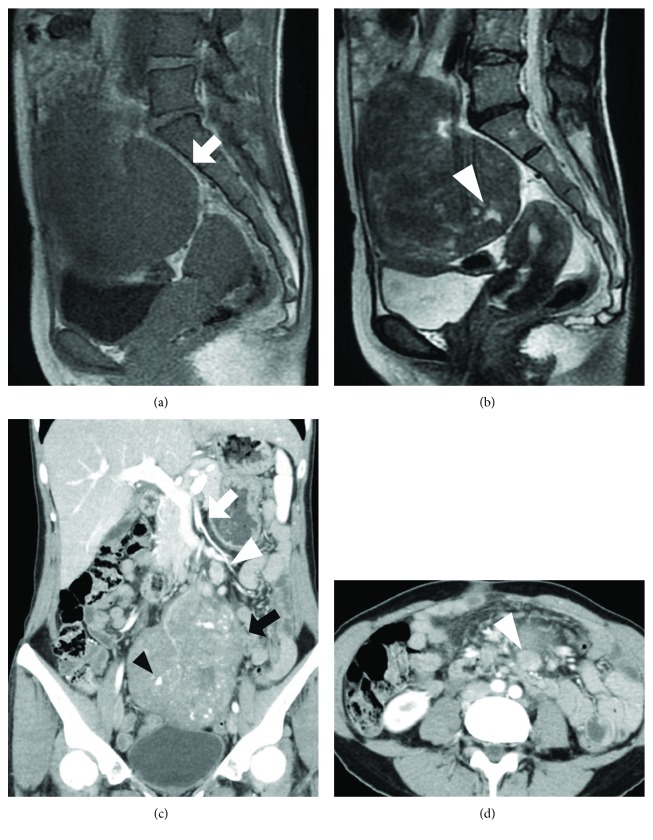
Abdominal MRI and abdominal contrast-enhanced CT. Abdominal MRI shows a homogeneous and well-demarcated 74 × 98 × 122 mm mass at the cranial part of the uterus (arrow), with a low signal intensity on T1-weighted image (a) and mostly low signal intensity and partially high intensity on T2-weighted image (b) (arrowhead). Abdominal contrast-enhanced CT shows a well-demarcated oval mass with a smooth margin and enhancement in the pelvis (black arrow). The enhanced arteries in the tumor are detected (black arrowhead). The tumor is supplied by the superior mesenteric artery (white arrow), and the surrounding lymph nodes are enlarged (white arrowhead) (c, d).

**Figure 2:**
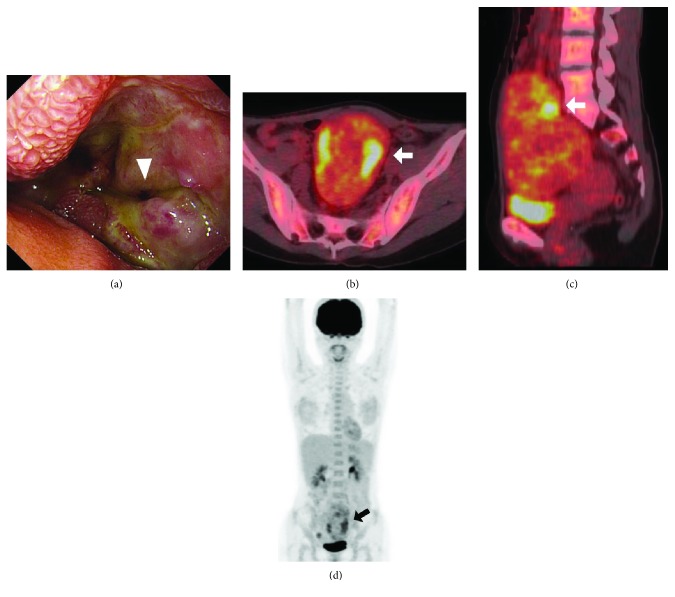
Upper gastrointestinal tract endoscopy and FDG-PET/CT. Upper gastrointestinal tract endoscopy shows a tumor with ulcer formation (arrowhead) in the third part of the duodenum, involving half the circumference (a). FDG-PET/CT axial image (b) and sagittal image (c) show heterogeneous uptake inside the tumor (white arrow) with SUVmax 6.3. FDG-PET/CT whole body image (d) also reveals heterogeneous uptake inside the tumor (black arrow).

The abdomen was flat and soft, with an elastic mass of poor mobility which was the size of an infant's head was palpable below the umbilicus to above the pubis. There were no blood test abnormalities; CEA, CA19-9, SCC, and the interleukin 2 receptor level were within normal limits. Abdominal MRI revealed a homogeneous and well-demarcated 74 × 98 × 122 mm mass near the cranial end of the uterus, with a low signal intensity on T1-weighted image, and mostly low signal intensity and partially high intensity on T2-weighted image (Figures 1(a) and 1(b)). Abdominal-enhanced computed tomography (CT) showed a well-demarcated and contrast-enhanced oval mass with a smooth margin in the pelvis. The tumor was supplied by the superior mesenteric artery, and the surrounding lymph nodes were enlarged (Figures 1(c) and 1(d)). Upper gastrointestinal tract endoscopy showed an easily bleeding tumor with an ulcer in the third part of the duodenum, involving half the circumference (Figure 2(a)). The biopsy results were inflammatory exudates and granulation tissues. FDG-PET/CT showed heterogeneous uptake inside the tumor with SUVmax 6.3 (Figures 2(b)–2(d)). A slight 18F-FDG uptake was observed in the enlarged lymph nodes with SUVmax 2.6. Along with the facts that there were no malignant cells detected by endoscopic biopsy and the CT image showing enlarged surrounding lymph nodes, the patient was suspected of nonepithelial malignancy of the duodenum such as gastrointestinal stromal tumor (GIST) and malignant lymphoma. As we could not make a definite diagnosis preoperatively, she underwent surgery for an accurate diagnosis and treatment.

**Figure 3:**
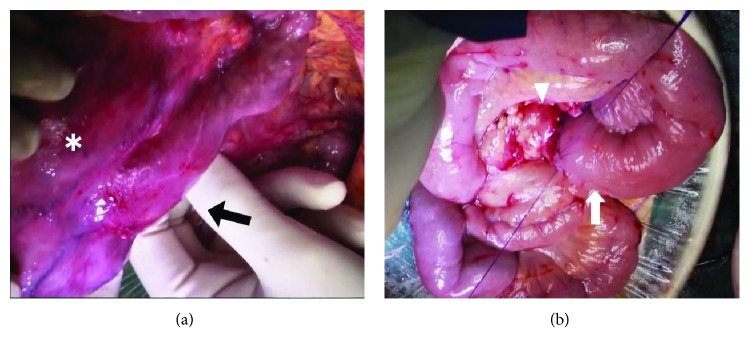
Surgical findings. Surgical findings show a 12 cm tumor mainly in the third part of the duodenum (asterisk); the duodenum and the ligament of Treitz (black arrow) are pulled in a caudal direction by the tumor (a). The horizontal part of the duodenum (arrowhead) is end-to-end anastomosed to the jejunal stump (white arrow) (b).

Surgery was performed under general anesthesia; laparotomy was performed via midline incision from 5 cm above the umbilicus to above the pubis. There was a 12 cm tumor centered on the third part of the duodenum, extending to the fourth part. The tumor extended into the pelvis and pulled the duodenum and the ligament of Treitz caudally (Figure 3(a)). There was no infiltration of the tumor into the pancreas or the small intestine. The duodenum was transected at the third part of the duodenum on the oral side. On the anal side of the tumor, the jejunum immediately after the ligament of Treitz was transected, and the tumor was resected. The enlarged lymph nodes sampling were also performed. In reconstruction, end-to-end duodenojejunostomy was performed (Figure 3(b)). She had an uneventful postoperative course and was discharged 9 days after surgery.

**Figure 4:**
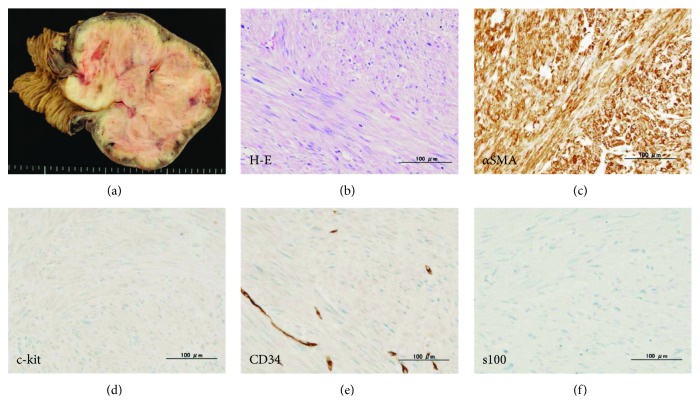
Pathological findings. Pathological testing shows a solid tumor growing from the muscle layer of the duodenum through the serous membrane (a). Hematoxylin and eosin staining show uniform growth of spindle cells in an intricate manner (b). On immunostaining, *α*-smooth muscle actin is positive (c), and S-100, c-kit, and CD34 are negative (d–f).

Pathological findings showed a solid tumor growing from the muscle layer of the duodenum through the serous membrane. The duodenal mucosa was invaginated at the site of the tumor, forming a deep ulcer (Figure 4(a)). The tumor showed uniform growth of long and spindle-shaped cells in an intricate manner, and the number of nuclear divisions was 1 or less per 50 visual fields (Figure 4(b)). There were no evidence of calcification, hemorrhage, or degeneration within the tumor. On immunostaining, *α*-smooth muscle actin was positive, while S-100, c-kit, and CD34 were negative (Figures 4(c)–4(f)). The MIB-1 index was low at 3% maximum, and there were no neoplastic lesions in the enlarged lymph nodes. Based on these findings, a leiomyoma was diagnosed. Since then, no relapse has occurred for two years and six months.

## 3. Discussion

Primary benign tumors of the duodenum are relatively rare. Darling and Welch [2] reported the frequency of primary benign tumor of the duodenum as 0.12% (21 of 17,070 autopsy patients), Ebert et al. [3] as 0.03% (8 of 25,000 autopsy patients), and Raiford [4] as 0.02% (13 of 56,500 operated and autopsy patients). The percentage of benign tumors of the duodenum among all benign tumors of the small intestine was 30% (35 of 115 patients) in the report by Darling and Welch [2] and 15% (208 of 1399 patients) in the report by River et al. [5], with slightly lower frequency than those in the jejunum and ileum. Among benign tumors of the small intestine, the frequency of primary leiomyoma is the highest after adenoma and lipoma, of which 25.3% are accounted to be duodenal primary [5, 6].

The second portion of the duodenum is the most common site affected by leiomyoma of the duodenum, accounting for more than 50% of all cases [7]. The most common clinical symptom is melena resulting from ulcer formation; abdominal pain, diarrhea, and constipation may also occur [7]. The incidence of extraductal growth is three times greater than that of intraductal growth; therefore, ductal obstruction is unlikely to occur [7]. It is presumed that this tumor was present when the patient was diagnosed with iron deficiency anemia 5 years prior to the current presentation. Possible reasons for nondiagnosis include the location of the tumor (centered in the third part of the duodenum) and absence of obstructive symptoms, probably due to extraductal growth.

Although FDG-PET/CT is widely performed for differential diagnosis of benign and malignant diseases, a gastrointestinal leiomyoma, which is a benign tumor, rarely shows uptake. A literature search of PubMed (1950–2015), using “leiomyoma” and “FDG-PET/CT” as keywords, found no report of leiomyoma of the duodenum showing increased uptake on FDG-PET/CT. There are reports of increased uptake on FDG-PET/CT in primary leiomyomas of the esophagus [8], stomach [9], and jejunum. However, it is unclear why increased uptake was observed in the gastrointestinal leiomyoma presented herein.

Increased uptake on FDG-PET/CT has been reported in leiomyomas of the uterus, and Chura et al. [10] stated that the uptake is associated with vascular growth in the tumor. Kitajima et al. [11] reported that there was a mildly positive correlation between the SUVmax of a leiomyoma of the uterus and the tumor size. In our patient, the tumor showed contrast enhancement on CT and had a large diameter of 12 cm, suggesting that increased uptake on FDG-PET/CT is associated with vascular growth and tumor size. The inflammation due to the invagination could also be one of the reasons of positive FDG uptake.

This patient was suspected of having a malignant lymphoma or GIST and underwent tumor resection alone without lymphadenectomy. Combined resection is needed when there is tumor infiltration into the surrounding organs; when the tumor involves the pancreas, pancreaticoduodenectomy may be required. In this patient, the tumor could be resected with partial duodenectomy because there was no infiltration into the pancreas or surrounding organs. Since the tumor was benign based on the pathological findings, the selected procedure was appropriate. However, when a tumor shows increased uptake on FDG-PET/CT, it is highly likely to be malignant. Therefore, considering intraoperative findings, selecting the appropriate surgical procedure is required to avoid excessive or insufficient surgery.

## 4. Conclusions

Increased uptake on FDG-PET/CT may be observed in a leiomyoma of the duodenum as seen in this patient. Therefore, a leiomyoma should be considered in the differential diagnosis of neoplastic diseases of the duodenum showing increased uptake on FDG-PET/CT.

## Abbreviations

MRI: Magnetic resonance imaging
FDG-PET/CT: 18F-fluorodeoxyglucose positron emission tomography
CT: Computed tomography
SUV: Standardized uptake value
GIST: Gastrointestinal stromal tumor.
